# The evaluation of the effect of performing guided lid surgery with enucleation of a cystic lesion; a case report

**DOI:** 10.1016/j.ijscr.2022.107385

**Published:** 2022-07-06

**Authors:** Aly Khaled Hussein Abdelazez, Hossam El-Dien Hany, Mohamed Ehab Gamal El Din, Mahmoued Medhat Mostafa El Meregy, Abdelrahman Mohamed Fahmy Abdelhameed, Ibrahim Mohammed El-Kabany, Abdulrahman Mostafa Abdelraouf, Mohamed Salah, Yasser Nabil El Hadidi, Mohammed Diaa Zein El Abdien

**Affiliations:** a Oral and Maxillofacial Surgery, Faculty of Dentistry, Ain Shams University, Egypt; b Faculty of Dentistry, Ain Shams University, Egypt; c Department of Oral and Maxillofacial Surgery, British University in Egypt, Egypt; d Department of Endodontics, Faculty of Dentistry, Ain Shams University, Egypt

**Keywords:** Guided lid surgery, Piezo-electric surgery, Cyst enucleation

## Abstract

**Introduction and importance:**

Dentigerous cysts are benign odontogenic cysts of developmental origin**.** Enucleation and marsupialization are still considered the blueprint of cystic lesion treatment**.**

**Case presentation:**

A 23-year-old male patient presented complaining of a minimal swelling in his upper jaw with slight tenderness in his upper anterior teeth. Cone Beam Computed Tomography (CBCT) on the maxilla was requested. The cystic lesion was found to be minimally expansile with intact cortical plates of the maxilla in the affected area. The CBCT was used to fabricate a cutting guide to determine the exact location of the bony window to fully access the lesion. Root canal treatment was done for the affected non-vital teeth. The cuts were done using a piezo-electric device. Complete enucleation was done for the lesion followed by fixation of the cortical bone lid using micro-plates and screws. The case was followed up after 6 months for new bone deposition using CBCT and 1 week, 1 month, and 6 months postoperatively for postoperative pain using the Visual Analogue Scale (VAS).

**Clinical discussion:**

Piezo-electric surgery was used due to the selective cutting merit to cut through bone while preserving the cystic lining intact. Lid surgery aims to maximize the volume of bone deposited in place of the defect by converting the cavity of the cystic lesion into a contained defect.

**Conclusion:**

Guided lid surgery using a piezo-electric device could be a useful technique for cystic enucleation regarding the new bone formation and pain level.

## Highlights

• Dentigerous cyst is the most common odontogenic cyst.
• Piezoelectric surgery offers minimal tissue trauma.
• Lid surgery preseves bone.
• Microplate fixation
• Odontogenic cyst management

## 1 Introduction

Dentigerous cysts, also known as follicular cysts, are benign odontogenic cysts of developmental origin [1]. Dentigerous Cysts are most commonly, but not exclusively, present in the mandibular third molar region accounting for about 20–30 % of all bone cysts in the head and neck region [2].

Often discovered by chance, dentigerous cysts are usually asymptomatic and can displace nearby structures and in extreme cases could lead to jaw fractures [3]. Although very rare, it has been reported that dentigerous cysts may arise from inflammatory origin specifically from infected predecessor teeth [4]. Dentigerous cysts affect the crowns of unerupted teeth and can undergo malignant transformation. The cyst and the causing tooth are usually removed concurrently [5].

Cystic lesions of the jaw have been under research for various treatment modalities. Yet, the procedures of enucleation and marsupialization are still considered the blueprint of cystic lesions treatment in the maxillofacial practice [6]. Complete elimination of the pathology, restoration of function, form, and esthetics along with the protection of adjacent vital structures and improving the patient's quality of life postoperatively are considered the main goals set by the surgeon during treatment planning [7]. Enucleation or curettage is still the most performed procedure for the treatment of cystic lesions of the jaws and is considered satisfactory for small lesions with a relatively simpler procedure with less morbidity and fewer post-operative complications as long as it is performed correctly, for larger lesions other options are proposed, such as decompression or marsupialization. For extremely large lesions with a known tendency for recurrence, resection has been the option with the most favorable results in terms of treatment but with larger morbidity factors [8].

The procedure of marsupialization can be used alone with the cystic cavity is sutured to the surrounding mucoperiosteum and bone deposition occurs and the lesion starts healing, or it can be used as a preliminary procedure to be followed by curettage after the lesion is smaller in size and easier to operate with fewer risks [9].

Treatment planning for cystic lesions has also taken great advantage of the progress and recent advances in maxillofacial radiology, where cone-beam computed tomography has been a great advent for the accurate estimation of the size, extension, and relation to neighboring structures of cystic lesions, which has brought a lot more success to treatment planning and accurate choice of the surgical technique [10].

## 2 Case presentation

**Fig. 1:**
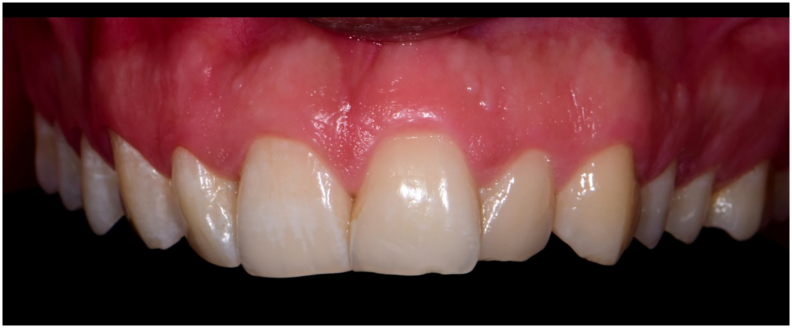
Pre-operative clinical photograph

The presented case report is recorded according to the SCARE checklist recommendations. A 23-year-old male patient presented complaining of a minimal swelling in his upper jaw with slight tenderness in his upper anterior teeth (Fig. 1). The patient is medically free with no significant drug history, no genetic background, or psychosocial history. The patient reported no history of dento-alveolar trauma.

By clinical examination, the teeth from the upper right 1 to the upper right 5 were found to be non-vital. Radiographic examination revealed a peri-coronal radiolucency related to an impacted supernumerary tooth with the apices of the upper right 1 to upper right 5 involved in the lesion. Aspiration biopsy was done revealing crystal clear yellowish cystic fluid. Cone Beam Computed Tomography (CBCT) on the maxillary arch was requested to further investigate the extent of the lesion and the exact position of the impacted supernumerary tooth.

The cystic lesion was found to be minimally expansile with intact buccal and lingual cortical plates of the maxilla in the area affected by the lesion. The intact cortical plates justified the use of the bone lid approach for the enucleation of the cystic lesion. The CBCT ensured that there is no communication with the maxillary sinus and the lesion is DE NOVO and not recurrent.

The CBCT was used to fabricate a cutting guide to determine the exact location of the bony window to be cut to fully access the lesion. The designed cutting guide was 3D printed in resin material before the surgical procedure. Root canal treatment was done in the non-vital teeth affected by the lesion.

**Fig. 2:**
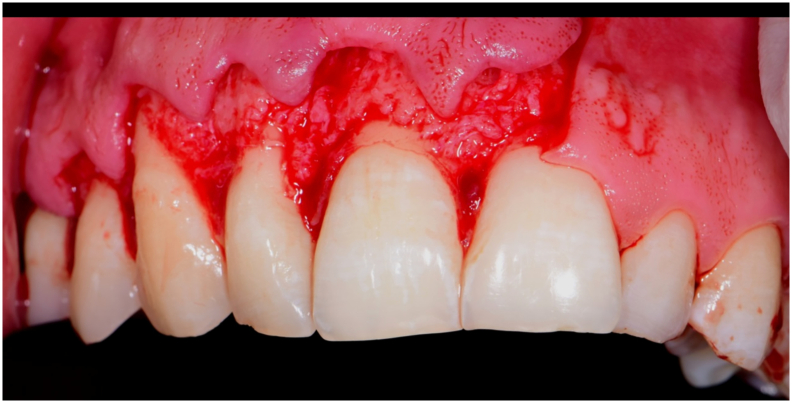
Full thickness muco-periosteal flap reflection

**Fig. 3:**
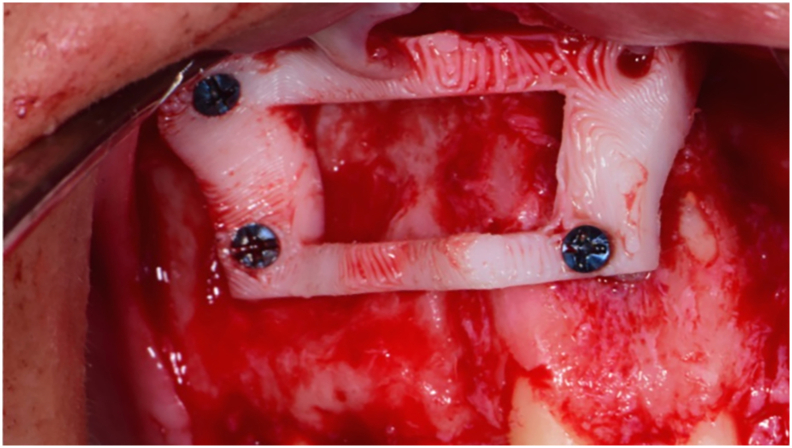
Fixation of 3D printed cutting guide

The surgical procedure was done under General Anesthesia based on the personal request of the patient. The surgical procedure was done by a consultant and a resident of oral and maxillofacial surgery practicing since 2017 similar cases. Endodontic therapy was performed by a specialist practicing endodontics since 2009. During the procedure, a full-thickness trapezoidal flap was done using a type 15 blade extending from the upper right 6 to the upper left 1 (Fig. 2). Reflection of the mucoperiosteum was done using a sharp mucoperiosteal elevator. The 3D printed cutting guide was fixed to the buccal cortical bone using self-drilling micro-screws of 5 mm length (Fig. 3). The osteotomy for the bone lid approach was done using a piezo-electric driven handpiece and the bone lid was carefully and bluntly dissected from the underlying cystic lining. The removed bone lid was placed in 0.9 % NaCl saline solution. After exposure of the lesion, the cystic lining was dissected from the surrounding bone using bone curettes of different sizes. Apicoectomy and retrograde filling using Mineral Trioxide Aggregate (MTA) were done for the endodontically treated teeth. Fixation of the removed bone lid was done using a 6-hole micro-plate of 1.5 mm thickness and 4 micro-screws of 5 mm length to achieve rigid fixation. The plate was planned to be removed only in case an infection developed around it. Flap closure was done using 4–0 Polyglycolic Acid (PGA) resorbable suture with a 3/8 cutting needle of 19 mm length.

The patient was prescribed Amoxicillin/Clavulanate 1 g tablets twice per day for 5 days, Ibuprofen 600 mg tablets twice per day for 5 days, and α-Chymotrypsin 2 tablets 3 times per day for 5 days. The patient strongly adhered to the postoperative instructions for the best outcome.

**Fig. 4:**
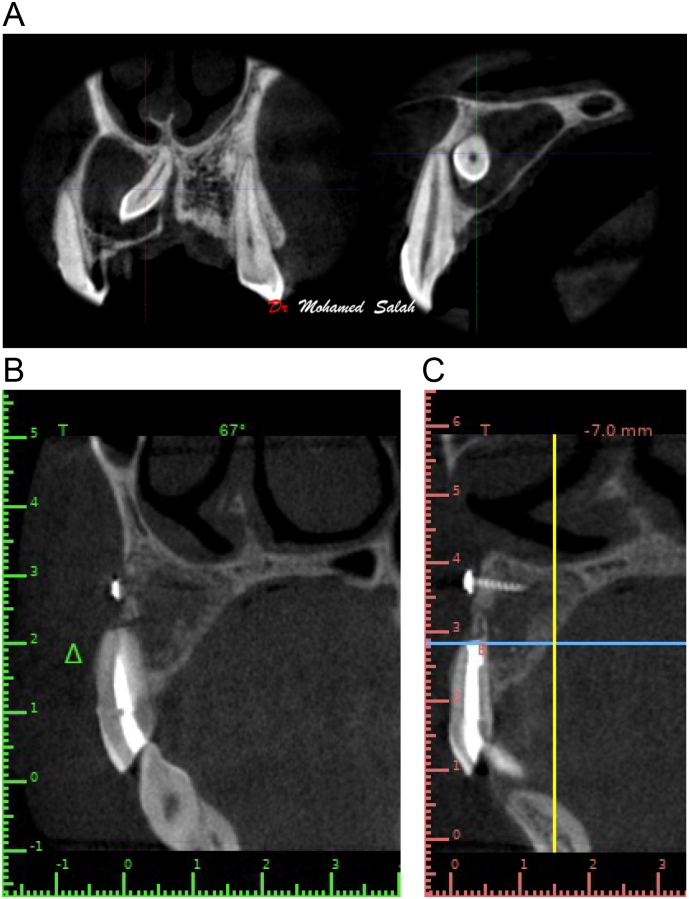
(A) Pre-operative CBCT. (B),(C) 6-month post-operative CBCT

The patient was scheduled for follow-up at 1 week, 1 month, and 6 months post-operative. A CBCT on the maxillary arch was requested during the 6-month follow-up visit to evaluate the amount of bone volume regained (Fig. 4A, B, and C). Post-operative pain was assessed using the Visual Analogue Scale (VAS) from 1 to 10 immediately post-operative and on each of the follow-up visits. In the follow-up, the lesion showed more than 90 % healing which doesn't usually take place in normal circumstances if conventional enucleating is done.

## 3 Discussion

Piezosurgery is a freshly introduced surgical maneuver for cutting hard tissue by targeting mineralized tissue and at the same time being gentle on the adjacent soft tissues such as the brain, nerves, vessels, and mucosa working based on ultrasound micro-vibrations [11]. Piezotome can be used in multiple oral and maxillofacial procedures such as dentoalveolar procedures (impactions and cyst removal), sinus floor elevation, alveolar ridge splitting, lateralization of the inferior alveolar nerve, bone graft harvesting, and orthognathic surgery, aesthetic facial surgery and temporomandibular joint surgeries [12].

The merits of using piezosurgery in oral surgery are the preservation of soft tissue and neurovascular bundles, the absence of vibrations making it more tolerable by the patient, and Improved survival of osteocytes compared to other bone harvesting methods leading to an earlier increase in BMP-4 and TGF-2 proteins and fewer pro-inflammatory cytokines in bone [13]. In cyst removal, either odontogenic or non-odontogenic, the use of piezoelectric derives showed more efficacy regarding bone healing after cyst removal and safety on the adjacent vital structures [14]. Despite the extended duration of the procedure, piezosurgery surpassed conventional surgery in terms of intraoperative bleeding, epithelial lining perforation, and also after surgery; complications, and recurrence [15].

Surgical guides have been introduced into the field of oral and maxillofacial surgery to eliminate drawbacks associated with complicated anatomy previously managed by the skill of the operator [16]. There are two types of guides: dynamic or static, the former provides the operator with a chance to apply the preplanned treatment to work while viewing it simultaneously on a screen while the latter is constructed using 3D printing using computed tomography or cone-beam computed tomography [17]. In oral and maxillofacial surgery templates have been involved in procedures including facial reconstruction secondary to trauma or pathology, orthognathic surgery, or implantology to help the operator perform bone cutting or drilling in the most accurate position and angulation or guide the condyle into its proper position [18]. Templates used as a guide for cutting using piezotome to create a bone lid have shown promising outcomes regarding the duration of operation and preservation of anatomic structure for removal of pathological lesions or impacted teeth [19]. Although templates play a role in the success of surgical procedures, they have been associated with some complications including high cost and extra time needed after processing [20].

The bone lid technique is another approach to the removal of mandibular lesions, where a window is cut within the bone using regular rotary instruments, micro–saws, or piezoelectric devices [21]. The bone removed is preserved and then placed again with mini plates, trans-fixation screws, or adhesive acrylic tissue. The advantage of using this technique is avoiding large bony defects after the removal of intra-bony lesions. Mini plates and screws are used to stabilize the bony window after placement and prevent fracture risk. This approach can also be used in the case of surgical endodontic treatment, removal of any fractured or failed implants, or deeply impacted teeth [22].

The article followed the guidelines recommended by the journal and followed the SCARE checklist advocated by Agha et al. [23].

## 4 Conclusion

Guided lid surgery using a piezo-electric device could be a useful technique for cystic enucleation regarding the new bone formation and pain level. A Randomized Clinical Trial (RCT) is to be conducted to compare this technique to the conventional technique for cystic enucleation.

## Consent

The patient perspective on the case was explained preoperatively and the patient fully accepted the treatment plan. The patient fully consented to surgical treatment and consented to the publishing of his photographic, histological, and radiographic data. Written informed consent was obtained from the patient for publication of this case report and accompanying images. A copy of the written consent is available for review by the Editor-in-Chief of this journal upon request.

## Funding

All authors confirm that the work is self-funded.

## Registration of research studies

Name of the registry: Clinical trial.org

Unique identifying number or registration ID: NCT05358275

## Ethical approval

Ethical Approval by Ethics Committee, Faculty of Dentistry, Ain Shams University

Reference number: 1078

## Provenance and peer review

Not commissioned, externally peer-reviewed

## Guarantor

Yasser Nabil El Hadidi

## CRediT authorship contribution statement

1) Aly Khaled Hussein Abdelazez, BDS
  Data Collection, Performing Surgical Procedure and Writing of Paper
2) Hossam El-Dien Hany, MDS, BDS
  Virtual Surgical Planning and Study Design
3) Mahmoued Medhat Mostafa El Meregy, BDS
  Data Collection and Writing of Paper
4) Abdelrahman Mohamed Fahmy Abdelhameed, BDS
  Data Collection and Writing of Paper
5) Mohamed Ehab Gamal El Din, BDS
  Data Collection and Writing of Paper
6) Ibrahim Mohammed El-Kabany, BDS
  Data Collection and Writing of Paper
7) Abdulrahman Mostafa Abdelraouf, BDS
  Data Collection and Revision.
8) Mohamed salah MDS, BDS
  Perforoming Endodontic Procedures and Photography.
9) Yasser Nabil El Hadidi, PhD, MOMSRCSEd, MDS, BDS
  Study Concept or Design, Data Collection, Data Analysis or Interpretation
10) Mohammed Diaa Zein El Abdien, PhD, MDS, BDS
  Study Case Supervision.

## Declaration of competing interest

The authors certify that they have no affiliations with or involvement in any organization or entity with any financial interest, or non-financial interest in the subject matter or materials discussed in this manuscript. The article is self-funded.

## Appendix A Supplementary data

SCARE Checklist Image 1
